# Analysis of the mechanical properties of wild type and hyperstable mutants of the HIV-1 capsid

**DOI:** 10.1186/s12977-016-0250-4

**Published:** 2016-03-15

**Authors:** Ruben Ramalho, Sanela Rankovic, Jing Zhou, Christopher Aiken, Itay Rousso

**Affiliations:** Department of Physiology and Cell Biology, Ben-Gurion University of the Negev, 84105 Beer-Sheva, Israel; Department of Pathology, Microbiology and Immunology, Vanderbilt University School of Medicine, Nashville, TN 37232 USA

**Keywords:** HIV, Capsid, Atomic force microscopy, Stiffness

## Abstract

**Background:**

The human immunodeficiency virus (HIV-1) capsid is a self-assembled protein shell that contains the viral genome. During the stages between viral entry into a host cell and nuclear import of the viral DNA, the capsid dissociates in a process known as uncoating, which leads to the release of the viral genetic material. Mutations that alter the stability of the capsid affect the uncoating rate and impair HIV-1 infectivity.

**Results:**

To gain further insight into the role of capsid stability during uncoating, we used atomic force spectroscopy to quantify the stiffness of the capsid. Empty in vitro assemblies of wild type (WT) and mutant recombinant HIV-1 capsid protein (CA) as well as isolated WT and mutant HIV-1 cores (i.e., filled capsids) were analyzed. We find that hyperstable CA mutant assemblies (A204C, A14C/E45C, E45A and E45A/R132T) are significantly stiffer than WT assemblies. However, the hardening effect of disulfide crosslinking (A204C and A14C/E45C) is lower than that of hydrophobic interactions (E45A and E45A/R132T).

**Conclusions:**

Our results demonstrate that mutations that increase the intrinsic stability of the HIV-1 capsid have an increased stiffness of their lattice.

## Background

The human immunodeficiency virus (HIV) is an enveloped retrovirus whose genetic material is initially in single-stranded RNA form. In mature viral particles, the viral genomic RNA is encapsidated within a cone-shaped capsid. The HIV capsid is a thin conical shell 100–120 nm in length that is formed during viral maturation by the assembly of about 1500 capsid protein (CA) molecules, which are organized into about 250 hexamers and 12 pentamers. It is widely accepted that the presence of pentamers induces the curvature necessary to form the cone shape of the capsid [1–3].

During infection, the viral RNA is reverse transcribed into double-stranded DNA and is then integrated into a host chromosome. Lentiviruses, such as HIV, infect non-dividing cells by traversing the nuclear pore as a nucleoproteic pre-integration complex that contains the viral DNA and integrase. Since the intact capsid is too large to cross the nuclear pore, capsid dissociation (uncoating) and HIV genome release are thought to occur prior to or during nuclear import. The state of the capsid during these steps is unclear, however, the capsid was demonstrated to play major roles, including evasion of innate immunity, motor protein recruitment for transport towards the nucleus [4], and mediation of nuclear import [5].

While the mechanism and timing of uncoating are presently unclear (see [5] and [6] for reviews), several studies suggest a correlation between capsid stability, uncoating, and viral infectivity. The cellular proteins transportin 3 (TNPO3) and cyclophilin A (CypA) were shown to interact with the viral capsid and to affect infection by modulating uncoating. In vitro data show that transportin 3 promotes uncoating whereas cyclophilin A can inhibit it [7, 8]. Several studies have shown that mutations in the CA protein affect capsid stability and, consequently, the uncoating rate [3, 9–12]. The mutated virus particles have significantly reduced infectivity and exhibit impairments in reverse transcription and virion trafficking. Interestingly, applying inhibitors to partially block reverse transcription gives rise to delayed uncoating [13, 14]. However, Xu et al. [15] suggested that the early stage of uncoating, which permits entry of a dye molecule into the capsid, is independent of reverse transcription. Nonetheless, the authors postulated a coupling between capsid uncoating and reverse transcription. Recent theoretical models [16] utilized the conversion of ssRNA into a dsDNA, which has a larger persistence length, as the mechanical driving force for sundering the capsid.

To assess the feasibility of any mechanical model of capsid uncoating, knowledge of capsid stiffness is essential. Previously, we have measured the mechanical properties of HIV-1 particles using nano-indentation measurements with an atomic force microscope (AFM) [17, 18]. Here, we apply the same nano-indentation method to measure the stiffness of empty capsids independently from the viral envelope. These empty capsids self-assemble from purified recombinant CA under high salt conditions in vitro [19]. In addition to measuring the stiffness of self-assembled wild-type capsids, we measured the stiffness values of assemblies of four hyperstable CA mutants. Comparing the wild type with these hyperstable mutants provides insight into the stabilizing effect of disulfide bonds (A204C and A14C/E45C) compared with hydrophobic interactions (E45A and E45A/R132T). Finally, we measured the stiffness of wild-type and E45A viral cores (i.e., of filled capsids) purified from virus particles. Our data demonstrate that mutations that delay HIV-1 uncoating also increase the physical stiffness of the HIV-1 capsid.

## Results

### CA assemblies form tubes and capsid-like cones

**Fig. 1:**
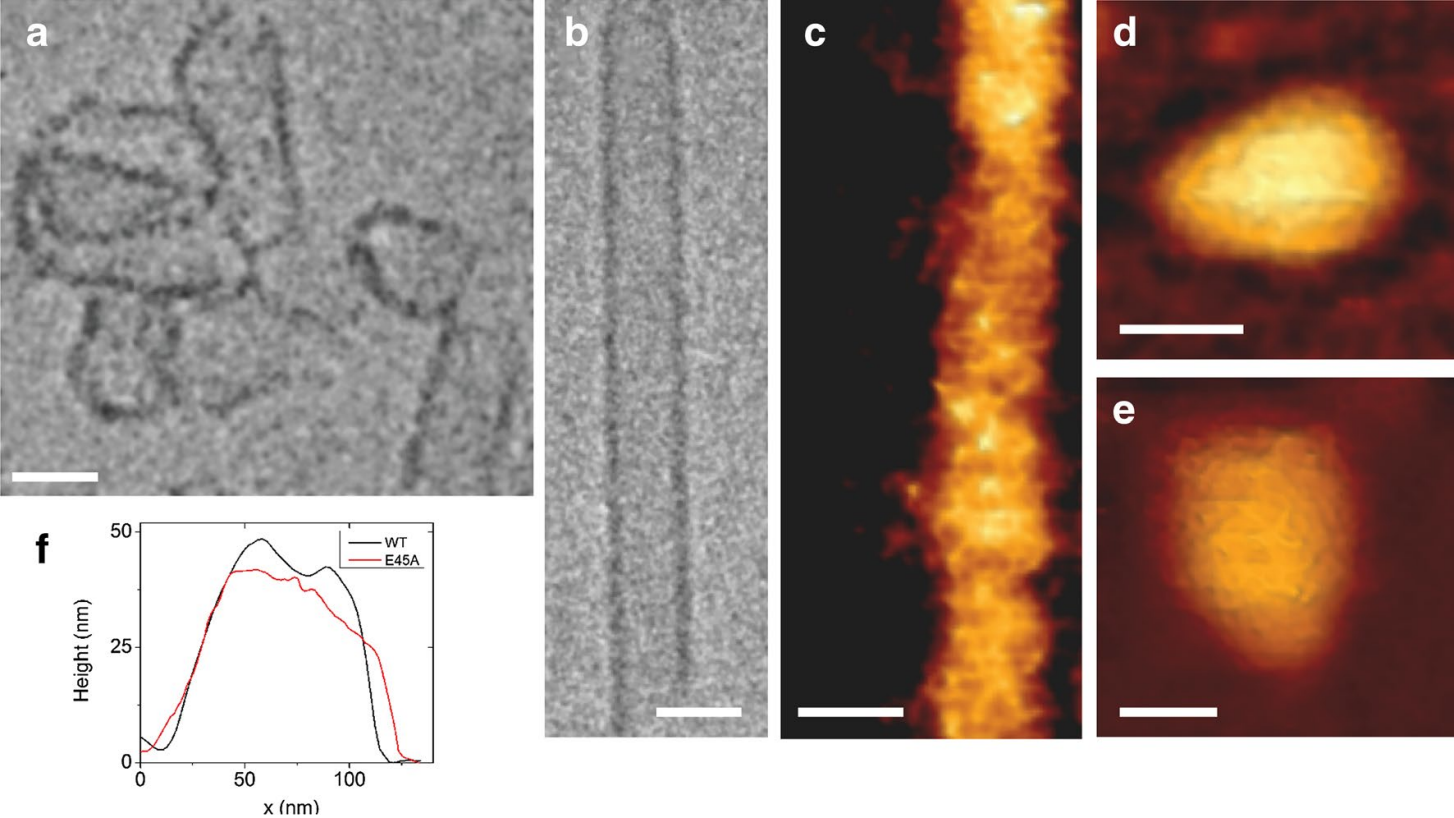
AFM and cryo-TEM images of recombinant capsid protein (CA) assemblies in vitro and isolated HIV-1 cores. **a** Cryo-TEM image of conical A204C CA assemblies. **b** CryoTEM image of tubular WT CA assembly. **c** Topographic AFM image of a tubular WT CA assembly. **d** Topographic AFM image of E45A conical CA assembly. **e** Topographic AFM image of isolated WT HIV-1 core. **f** Cross-sectional analysis of WT and E45A cores, *black* and *red lines*, respectively. AFM images were acquired in quantitative imaging (QI) mode. *Scale bars* are 50 nm

**Fig. 2:**
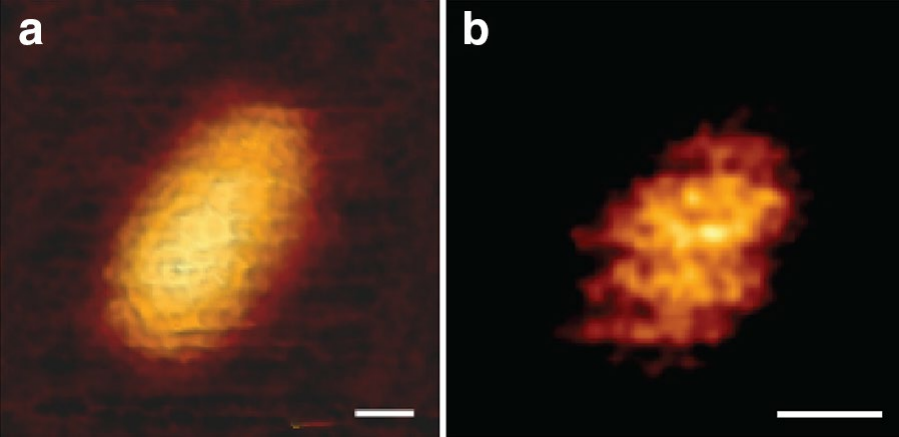
High resolution topographic imaging of WT conical CA assemblies using quantitative imaging (QI) mode AFM. **a** An image of the surface of a conical capsid revealing a honeycomb lattice on its surface. **b** An enlarged AFM portion of the top region of a conical CA showing the capsid lattice structure. *Hexagonal ring* structures (diam., ~10 nm) are detected. *Scale bars* are 20 nm

Purified recombinant CA protein was assembled into capsids under high-salt conditions and their structures were visualized by topographic AFM (Figs. 1, 2). WT CA assemblies form tubes (Fig. 1c) and cones (Fig. 2a), as previously described [3]. The same two forms were found for hyperstable mutant CA assemblies: E45A (Fig. 1d) and E45A/R132T, A204C and A14C/E45C (not shown), with varying relative abundances of tubes and cones. Tubes were of variable length (200 nm to tens of mircometers), diameter (typically 50–200 nm), and height (20–30 nm). Cone lengths were also variable, with the most common forms being 100–120 nm in length, although a few conical assemblies were ~200 nm in length. Cones were 80–100 nm wide at their broad end and 20–35 nm or 30–50 nm high, for capsids or isolated cores, respectively. Cryo-TEM was used to confirm the existence of both forms (Fig. 1a, b).

While the hyperstable mutant assemblies maintain their structure for 5–10 days without any detectable damage, WT CA assemblies were the least stable, being visibly disassembled after 48 h. Therefore, all measurements were performed on freshly assembled samples.

In order to compare the in vitro CA assembly with native viral capsids, HIV-1 cores were purified and their structures were visualized by AFM. Cores are, as described, conical in shape, with lengths of 100–120 nm, widths of ~90 nm, and heights of 20–40 nm. No difference in shape was apparent between WT (Fig. 1e) and E45A (not shown) cores. A cross-sectional analysis of representative WT and E45A cores is shown in Fig. 1f. Cores appear to be wider than expected due to convolution between the sample and the AFM probe.

Using sharp AFM tips, we were able to resolve the capsid honeycomb lattice on the surface of the CA assemblies (Fig. 2a). To visualize individual CA hexamers, we zoomed onto the top area of the capsid (Fig. 2b) where the curvature is minimal. In this relatively flat region, the features of the individual hexamers became evident. Measured hexamer diameter was approximately 10 nm, which is consistent with dimensions obtained by other methods [3].

### Hyperstable CA mutations increase capsid stiffness

**Fig. 3:**
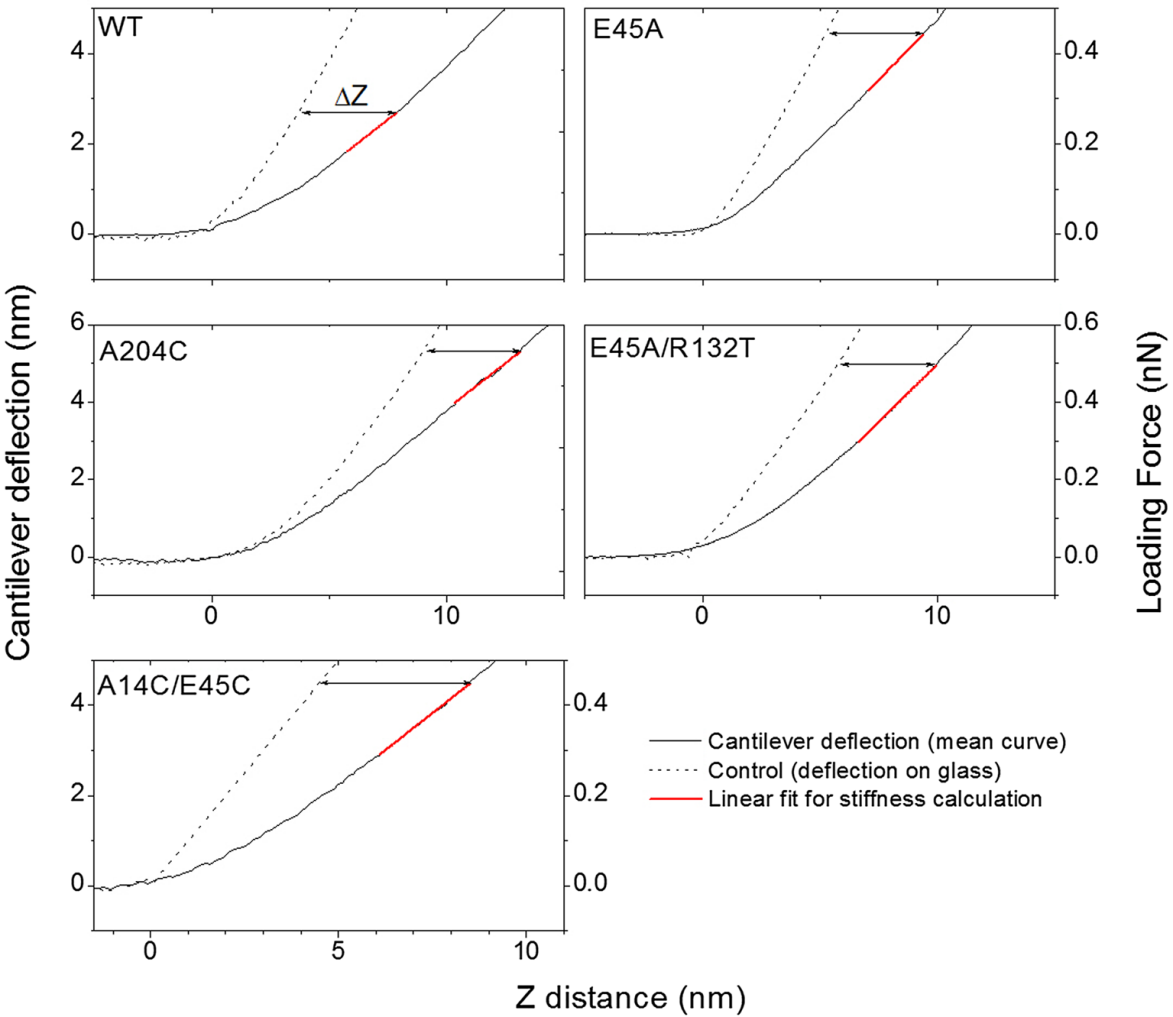
Representative averaged force–distance curves for CA assemblies (*solid lines*). The cantilever deflection curves (*dotted lines*) were obtained by acquiring force–distance curves of the glass substrate. All stiffness analyses were calculated at a maximal loading force that corresponds to 4 nm indentation depths (*double arrowhead line*). Stiffness values were calculated by fitting a linear function to the force curve region bounded by 3 and 4 nm indentation depths. The corresponding *line* fit is plotted in *red*

**Fig. 4:**
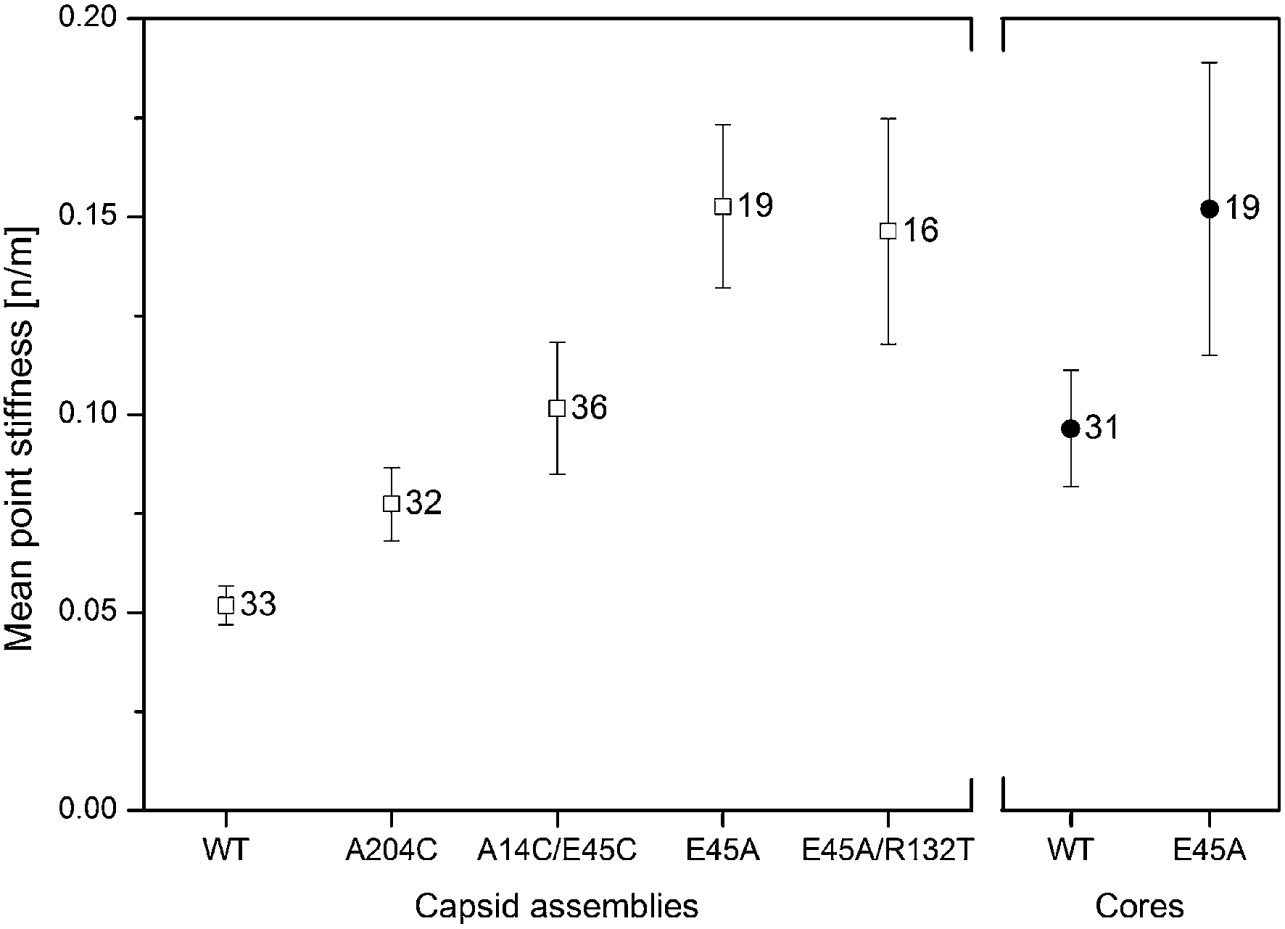
Averaged measured point stiffness of recombinant CA assemblies (*empty squares*) and isolated HIV-1 cores (*solid circles*). Each value was calculated as the average of ~100 force–distance curves obtained from individual capsids. The average stiffness values derived from all of the measurements were plotted. *Error bars* represent the SE of the mean. The number adjacent to each data point indicates the number of capsid particles analyzed

To measure the point stiffness of WT and mutant CA assemblies and of isolated viral cores, the AFM was operated in the nano-indentation mode. For the mechanical analysis only conical capsid assemblies were included. A representative averaged force–distance curve for each sample is shown in Fig. 3. The measured point stiffness values are summarized in Fig. 4. Uncertainty values represent the standard error of the mean. WT CA assemblies have the lowest averaged stiffness values of 0.052 ± 0.005 N/m (*n* = 33). Interestingly, isolated WT cores are almost two-fold stiffer than their in vitro-assembled counterpart, with an averaged stiffness value of 0.097 ± 0.015 N/m (*n* = 31, Mann–Whitney U p < 0.05). We then measured two CA variants that contain the E45A mutation. This mutation increases the hydrophilic interactions between adjacent CA proteins within the same hexamer. We observed that the E45A mutation produced the stiffest capsid structures, with similar values for E45A CA assemblies (0.153 ± 0.02 N/m, *n* = 19) and for isolated cores (0.152 ± 0.037 N/m, *n* = 19). The double mutant E45A/R132T CA assemblies exhibited stiffness values of 0.146 ± 0.029 N/m (n = 16), which is similar to the stiffness of the single mutant. The final pair of CA mutations introduces covalent crosslinking via the addition of disulfide bonds. The A204C mutation introduces a crosslink between CA hexamers, whereas the A14C/E45C double mutation introduces a crosslink between CA monomers within the hexamer. The stiffness values for both mutants are, 0.077 ± 0.009 N/m (n = 32) and 0.102 ± 0.017 N/m (*n* = 36), for A204C and A14C/E45C, respectively. Interestingly, the effect of the additional hydrophobic interactions (from E45A-containing mutations) on the stiffness of the assembled CA is larger than that of the covalent disulfide bridge (from the A204C and A14C/E45C mutations). This difference is statistically significant using the Mann–Whitney U test for p < 0.05.

## Discussion

The stiffness value of the empty WT HIV-1 capsid shell (assembled from recombinant CA) is significantly lower than that of a filled WT capsid (isolated viral core) or an enveloped virion [17, 18, 20]. The stiffness of the empty capsid is comparable to that of the empty capsid of a non-enveloped ssRNA virus, such as the norovirus [21]. In contrast, empty capsids of icosahedral DNA viruses, such as the enveloped herpes simplex virus 1 [22] and the non-enveloped mouse minute virus [23], are six- to ten-fold stiffer than empty capsids of WT HIV-1. Double-strand DNA is a much stiffer polymer than single-strand DNA or RNA, and is thought to exert high pressures on the capsid wall [16, 24, 25]. This suggests that capsid mechanical properties are highly adapted to the nature of their contents, as previously suggested [26].

The mechanical properties of retroviral capsids are also modulated by their contents, as we observed by comparing WT HIV-1 cores with recombinant WT CA assemblies. The RNA–protein complexes within the core are not thought to occupy the entire volume of the capsid [16], which rules out a potential contribution of internal pressure to capsid stiffness. Instead, we hypothesize that the capsid contents and the capsid interact in a manner that reinforces shell stiffness. Similar stabilizing interactions have been described in DNA viruses [27]. In contrast, this reinforcement was not observed in the case of the E45A mutant: the stiffness value for E45A CA assemblies was nearly identical to that of its isolated native core. This is probably because the empty capsid is relatively stiff, such that the capsid’s contents make no apparent contribution.

AFM analysis requires adsorption of the sample to a substrate which often deforms its structure. It is therefore possible, that the deformation of WT HIV-1 core increases the packing density of its contents, which leads to elevated core stiffness values compared with its WT CA counterpart. To address this possibility, we estimated the extent of deformation of the adsorbed core. The maximal thickness of an HIV-1 core is approximately 50 nm [1]. In our AFM analysis, the measured height of isolated cores is 35–50 nm, indicating that adsorption deforms the cores to only a relatively small extent. In addition, the contents of HIV-1 core are thought to occupy only about 20 % of their volume [16]. Taken together, the small deformation and low content occupancy suggest that the apparent reinforcement of WT cores is unlikely to be due to an increase in the packing density of their content.

Stiffness can be defined as the force needed to elastically deform a structure. In multimeric assemblies, such as capsids, this is intrinsically connected to the strength of the interactions between the building blocks and between the monomers within each building block. To explore the effect of inter- and intra-hexamer interactions on the stiffness of the capsid, we studied a series of four capsid-stabilized CA mutants. Mutations E45A and E45A/R132T increase hydrophobic intra-hexamer interactions, the double A14C/E45C mutation introduces covalent intra-hexamer crosslinking via a disulfide bond, while A204C introduces inter-hexamer disulfide crosslinking. We found that all four mutations elevate capsid stiffness in comparison with that of the assembled WT capsid. Interestingly, increasing intra-hexamer interactions resulted in a larger increase in capsid stiffness than from increasing inter-hexamer interactions (Fig. 4). In addition, we observed that increasing the hydrophobic interactions between CA monomers has a significantly larger effect on capsid stiffness than increasing covalent crosslinking (compare Fig. 4 A14C/E45C to E45A and E45A/R132T). To explain this result one has to consider the orientation of the applied force compared to the above monomers interactions. If the force is being applied along the bond axis then covalent bond is expected to be stronger than hydrophobic interactions. However, to measure the stiffness of the capsids we apply a force which is perpendicular to the monomer–monomer interactions axis. Such a perpendicular force does not act on the bond itself, but rather on the interactions between monomers’ interfaces which are mostly hydrophobic. Hence, our results imply that the applied force is acting on interactions between these hydrophobic interfaces and therefore stronger hydrophobic interactions lead to greater stiffness.

Capsid stability has been previously assessed biochemically by assembly efficiency and by comparing dissociation over time [3, 11, 28, 29]. According to those analyses, the capsid disulfide crosslinking mutants, A204C and A14C/E45C, are the most stable of the four mutants analyzed in our study. This apparent discrepancy between the above result and our findings indicates that biochemical stability does not strictly correlate with mechanical stiffness, as has been previously shown [30].

Finally, the E45A/R132T double mutant has previously been shown to partially restore viral infectivity lost following the original E45A mutation while maintaining the latter’s stability in vitro [29]. The authors suggested that the mutation instead restored the capsid’s interactions with cellular factors, or the consequences thereof. In support of their hypothesis, we find that the stiffness of E45A/R132T mutant CA assemblies is similar to that of the E45A mutant.

## Conclusions

We present an AFM analysis of the morphology and mechanical properties of the HIV-1 capsid. The topographic AFM images of CA assemblies and cores are in good agreement with TEM-resolved structures.

Using self-assembled capsid protein structures allowed us to measure capsid stiffness in isolation from its contents, which revealed that the empty WT capsid is extremely soft and that its core contents have a reinforcing effect on it. This finding of an extremely soft capsid fits well with uncoating models [16] in which DNA synthesis mechanically destabilizes the capsid up to a breaking point.

As our results show, capsid-stabilizing mutations significantly stiffen the capsid structure, which, as Forshey et al. [11] showed, results in decreased infectivity in several of these mutations. The higher stiffness of the native WT HIV-1 cores compared to the corresponding recombinant CA assemblies suggests that the contents of the viral core buttresses the capsid walls. This suggests, in turn, that the rigidification of the viral genome that occurs during reverse transcription may provide a trigger for uncoating, as suggested from previous uncoating studies in cells [13, 14, 31]. Nevertheless, capsid stiffness is not strictly correlated with infectivity, as shown by the phenotype of the E45A/R132T double mutant. Future work will test the effects of reverse transcription and cellular factors on capsid stiffness and uncoating in vitro.

## Methods

### Capsid assembly

Purified recombinant WT and mutant HIV-1 capsid proteins (E45A, E45A/R132T, A204C and A14C/E45C) were restored from lyophilized form by resuspension in storage buffer (20 mM Tris–HCl at pH 8.0, 40 mM NaCl, 60 mM β-mercaptoethanol) to a final concentration of 160 μM and self-assembled using a method previously described [3]. Briefly, the resuspended CA was dialyzed against CAB (capsid assembly buffer: 100 mM Tris–HCl at pH 8.0, 200 mM NaCl) overnight at 4 °C in mini dialysis cups (Slyde-A-Lyzer MINI, Thermo Scientific). The resulting CA assemblies were then characterized using AFM and cryo-transmission electron microscopy (cryo-TEM).

### Viral core purification

Pseudovirion particles were produced by transfection of human embryonic kidney (HEK) 293T cells with the ΔEnv HIV-1 genome vector containing either the WT or E45A CA mutant sequence, using a method previously described [32]. Briefly, cells were transfected using polyethylenimine (branched, MW ~25,000, Aldrich), the medium was changed after 20 h and the supernatant containing pseudoviral particles was collected 26 h post-transfection. The supernatant was centrifuged for 10 min at 1000×*g*, filtered through a 0.45 μm pore filter, and centrifuged over an OptiMEM (Sigma) cushion at 100,000×*g* for 2 h at 4 °C. Viral pellet was resuspended in TNE (100 mM NaCl, 0.1 mM EDTA, 50 mM Tris–HCl pH 7.4) and concentrated using a Vivaspin 20 column (100,000 MWCO, Sartorius). Viral cores were purified by mixing an aliquot of purified and concentrated HIV-1 pseudoviral particles with an equal amount of 1 % Triton X-100 in 3-(N-morpholino)propanesulfonic acid (MOPS) buffer (200 mM NaCl, 100 mM MOPS, pH 7.0). The mixture was incubated for 2 min at 4 °C and cores were spun down at 13,800×*g* for 8 min. Supernatant was gently removed and pelleted cores were washed twice by adding 80 µL of MOPS buffer and centrifuging at 13,800×*g* for 8 min. The pellet was resuspended in 10 µL MOPS by pipetting and the resulting cores were characterized using AFM.

### Atomic force microscopy (AFM)

For preparation of AFM samples, 10–20 μL of solution containing capsid assemblies or cores was deposited on hexamethyldisilazane- (HMDS-) coated microscope glass slides, incubated for 15–30 min at room temperature, rinsed, and measured in buffer (CAB for capsid assemblies and MOPS for cores).

Measurements were carried out with a JPK Nanowizard ULTRA Speed AFM (JPK Instruments, Berlin, Germany) mounted on an inverted optical microscope (Axio Observer, Carl Zeiss, Heidelberg, Germany). Silicon nitride probes (mean cantilever spring constant, k_cant_ = 0.1 N/m, DNP, Bruker) were used for stiffness measurements, and sharp silicon probes (mean k_cant_ = 0.07 N/m, MSNL, Bruker) were used for high-resolution imaging. Topographic imaging was performed in quantitative imaging (QI) mode, which is a force-curve based imaging mode.

Capsid stiffness was determined based on indentation type experiments as previously described [18]. Briefly, 100 force–distance (F–D) curves were obtained for each point stiffness measurement at a rate of 20 Hz. Each individual F–D curve was acquired by elastically indenting the sample to a maximum of 4 nm (corresponding to a maximum force of 0.2–1.5 nN). Prior to analysis, each curve within a set was shifted to set the deflection in the non-contact section to zero. The set of F–D curves was then averaged. Stiffness was derived mathematically from the slope of the force distance curve. A linear function was fitted to a region of the loading part of the force-distance curve bounded by 3 and 4 nm indentation depths. Averaged force-distance curves were converted from deflection units (V) to a loading force (N) by multiplying the former by the deflection sensitivity (in nm/V, derived from a force-distance curve performed on glass) and the spring constant (N/m) of the cantilever. The measured stiffness comprises the stiffness constants of both the capsid (k_CA_) and the cantilever (k_cant_). The stiffness of the capsid was computed according to Hook’s law on the assumption that our experimental system can be modeled as two springs arranged in series. To reduce error in the calculated point stiffness, we chose cantilevers such that the measured point stiffness was less than 70 % of the cantilever spring constant. Statistical differences between stiffness means were tested using parametric (ANOVA) and nonparametric (Kruskal–Wallis) statistics at a level of ≤0.0001. Pairwise comparisons were done using the nonparametric Mann–Whitney U statistical test.
